# Protective effects of carotenoids against blue light induced-cellular damage in human retinal pigment epithelium

**DOI:** 10.1007/s10068-024-01757-z

**Published:** 2025-01-27

**Authors:** Jong Hoon Won, Dmitri Sitnikov, Jina Hong

**Affiliations:** 1 Amway Corporation, Amway I&S, 7575 Fulton St E, Ada, MI 49355 USA; 2 Nutrilite Health Institute, Amway I&S, 5600 Beach Blvd, Buena Park, CA 90621 USA

**Keywords:** Blue-light, Oxidative stress, DNA damage, Cellular senescence, ARPE-19

## Abstract

The retinal pigmented epithelium (RPE) is constantly exposed to visible light, including blue light (BL) that creates reactive oxygen species (ROS), which are harmful to DNA and induce cellular senescence. Carotenoids are recognized for their antioxidant properties, but their protective effect on DNA repair and cellular senescence under BL induced oxidative stress has not been evaluated. After BL irradiation, the positive senescence-associated-β-galactosidase (SA-β-gal) staining, and gene expression of *p16*^*INK4a*^ and *p21*^*Waf/Cip1*^ were upregulated in ARPE-19 cells. Pretreatment with carotenoids reduced ROS, p-H2A.X nuclear foci, and SA-β-gal positive cells induced by BL irradiation. Furthermore, pretreatment with carotenoids reduced the secretion of IL-6 and VEGF triggered by BL. Since increased senescent cells and secretion of IL-6 and VEGF are involved in age-related macular degeneration pathogenesis, our results support that carotenoid supplementation has a potential role in protecting the eyes from the deleterious effects of excessive BL exposure.

**Supplementary Information:**

The online version contains supplementary material available at 10.1007/s10068-024-01757-z.

## Introduction

Blue light (BL) is part of the visible light spectrum with a wavelength range between 400 and 495 nm (Zou and Dai, 2015). BL can reach the retina and is considered to have the potential to cause phototoxic retinal damage since it has high energy that can generate reactive oxygen species (ROS) (Godley et al., 2005). It has been considered that BL irradiation may induce retinal damage and contribute to the pathogenesis of age-related macular degeneration (AMD) (Alaimo et al., 2019). Oxidative stress, apoptosis, and inflammation of retinal pigment epithelium (RPE) play crucial roles in both the onset and progression of AMD (Abu-Amero et al., 2016).

Cellular impairment, including organelle and DNA damage, can induce cellular senescence (Herranz and Gil, 2016). Cellular senescence events such as cell-cycle arrest, as well as prolonged senescence may lead to chronic inflammation, cardiovascular disease, and AMD (Kozlowski, 2012).

The RPE, in the outer retina, is subjected to high levels of oxygen and abundant light influx including BL, rendering it highly vulnerable to oxidative stress. This excessive burden of oxidative stress invokes the onset and progression of several retinal diseases (Strauss, 2005).

Carotenoids, a group of pigments contained in various fruits and vegetables, play a drastic role in promoting eye health because of their antioxidant properties and other beneficial effects (Khachik et al., 2002). Approximately 40 natural carotenoids are commonly ingested through the human diet; however, only 15 to 20 of these are commonly detectable in human serum and tissues, including lycopene, β-carotene, lutein, and zeaxanthin (Khachik et al., 2002). Lutein and zeaxanthin are found in high concentrations in the macula of the eye and provide macular protection through their antioxidant and light-filtering properties (Kotagiri et al., 2022). An inverse correlation between AMD and macular pigment density has long been established (LaRowe et al., 2008).

Previous studies demonstrated the protective effects of carotenoids such as lutein and zeaxanthin against H_2_O_2_-induced apoptosis in ARPE-19 cells (Leung et al., 2020; Liu et al., 2017). Additionally, Lutein was evaluated for its antioxidant properties in mitigating oxidative stress caused by BL irradiation (Alaimo et al., 2019). However, despite these efforts, the protective effect of carotenoids on reducing DNA damage and cellular senescence in damaged RPE under BL exposure conditions remains poorly understood.

Here using ARPE-19, a human RPE cell line derived from normal eyes, we provide evidence that the carotenoids – lycopene, lutein, zeaxanthin, and β-carotene – protect against the cellular damage induced by BL irradiation. We analyzed ROS levels, p-H2A.X levels as a marker of DNA damage, and senescence-associated β-galactosidase (SA-β-gal) activity as a marker of cellular senescence. We also explored the underlying mechanisms of antioxidant effects and DNA repair. Our study revealed the protective roles of carotenoids on the BL-induced RPE damage model.

## Materials and methods

### Materials

Β-carotene, lycopene, lutein, zeaxanthin, tetrahydrofuran, dimethyl sulfoxide, ethyl lactate, and ceramic balls (2.8 mm, Omni international) were purchased from Sigma Aldrich. An LC–MS grade water, Formic acid (FA), Methanol (MeOH), and Methyl-tertbutyl ether (MeMT) were obtained from Fisher Scientific. Dulbecco’s Modified Eagle’s Medium/Nutrient Mixture F-12 medium was obtained from ATCC. Fetal bovine serum (FBS) was obtained from HyClone (Logan, UT). The human IL-6 DuoSet ELISA kit was obtained from R&D Systems (Minneapolis, MN). Senescence-associated β-galactosidase assay kit (Cat# 9860 and 23,833), anti-HDAC2 antibody (Cat# 5113), and anti-GAPDH antibody (Cat# 2118) were obtained from Cell Signaling Technology (Danvers, MA). anti-OGG1 antibody (Cat# PA131402) purchased from Fisher. Anti-Nrf2 antibody (sc-365949) was obtained from Santa Cruz Biotechnology (Dallas, TX). CellTag™ 700 and IRDye® 800CW Goat anti-Rabbit IgG secondary antibody was obtained from LI-COR (Lincoln, NE). CellROX™ was purchased from ThermoFisher. blue LED light was obtained from RubyLuxLights. All other reagents were obtained from Sigma. Antioxidant assay kit (Cat#709,001) was purchased from Cayman Chemical. Lycopene was purchased from Lycored (Mehoz Hadarom, Israel); lutein and zeaxanthin were purchased from DSM (Alsace, France); β-carotene was purchased from BASF Australia (Victoria, Australia). All extractions underwent verification via vendor certification.

## Cell culture

The ARPE-19 cells were maintained in DMEM: F12 Medium including 10% fetal bovine serum (FBS), penicillin (100 U/mL), and streptomycin (100 μg/ml) at 37 °C in a humidified atmosphere with 5% CO_2_. Cells were passaged by trypsin–EDTA (0.05%) solution every 2–3 d.

## MTT assays

ARPE-19 cells (5 × 10^4^ cells/well) were seeded into 96-well plates. The carotenoids were dissolved in DMSO and tetrahydrofuran (THF) in a ratio of 2:1 (v/v). After incubation for 24 h, the media was replaced with media containing Lutein/zeaxanthin (Lu/Zea), lycopene, or β-carotene at concentrations ranging from 0 to 10 µg/ml. 24 hours later, 5 µL of MTT reagent (20 mg/ml) was treated and the plates were incubated for 1 h. Following this incubation, the media was aspirated and DMSO was added. Absorbance was analyzed at 570 nm using a microplate reader (Spectramax M5, Molecular Devices, CA, USA).

## Blue LED light irradiation

ARPE-19 cells (5 × 10^4^ cells/well) were seeded onto a black 96-well plate and maintained at 37 °C for 24 h. The cells were treated with carotenoids at a concentration of 400 µg/mL for Lu/Zea or 745 µM for lycopene and β-carotene for 48 h. The N-acetylcysteine (NAC, 1 mM) was used as a positive control. After the pretreatment, CellROX™ (ThermoFisher) was added to the cells, and the cells were irradiated with 2.5 mW/cm^2^ of blue LED light at wavelength 410 nm for 90 min. Fluorescence readings were taken at 485/520 nm in a multi-well plate reader. For Immunocytochemistry, the BL exposed cells were fixed with 4% paraformaldehyde and washed in PBS at RT. Fluorescence images were taken using a fluorescence microscope. Cells were treated with carotenoids at a concentration of 10 µg/ml of Lu/Zea, lycopene, or β-carotene for 48 h.

## Immunofluorescence

The cells in an 8-chambered slide (Ibidi, Gräfelfing, Germany) were fixed with 4% paraformaldehyde (PFA), and made permeable using 0.1% tritonX100 in PBS, and then blocked with 5% BSA in PBS for 1.5 h. The cells were incubated with p-Histone H2A.X (Ser 139) antibody (Santa Cruz, sc-517348, dilution ratio is 1:400) at 4 °C overnight. The cells were washed with PBST, and the cells were incubated with Alexa Fluor 488-conjugated (1:500; ThermoFisher) antibody diluted in 3% BSA in PBS at room temperature in the dark for 1 h. 4′,6-diamidino-2-phenylindole (DAPI) was used to counterstain the cell nuclei and then imaged by fluorescence microscope (Leica DMI6000B, Leica Microsystems, Wetzlar, Germany).

## Isolation of RNA, cDNA synthesis, and real-time PCR

Total RNA was isolated from the cells using the RNAeasy kit from Qiagen following the manufacturer’s manual. cDNA was synthesized with 2 µg of isolated RNA using iscript cDNA synthesis kit (Biorad). Real-time PCR was performed with Bio-Rad CFX 96 real-time PCR detection system using SsoFast™ EvaGreen® Supermix. PCR specificity was determined by melting curve analysis. A reference gene (GAPDH) was used as an internal standard. Gene-specific primers used are NQO1 Forward 5′-TGGCTAGGTATCATTCAACTC-3′; NQO1 Reverse 5′-CCTTAGGGCAGGTAGATTCAG-3′; HO-1 Forward 5′-GCCAGCAACAAAGTGCAAGAT-3′; HO-1 Reverse 5′-GGTAAGGAAGCCAGCCAAGAG-3′; Sod1_Foward 5′-GCCAAAGGATGAAGAG-3′; Sod1_Reverse 5′-CCACAAGCCAAACGAC-3′; Gclc_Forward 5′-TTGGAGACCAGAGTATGGGAGT-3′; Gclc_Reverse 5′-CTGGGAAATGAAG TTATTGTGC-3′; Gapdh_Forward 5′-ACCACA GTCCATGCCATCAC-3′;Gapdh_Reverse 5′-TCCACCACCCTGTTGCTGTA-3'; p16^INK4A^_Forward 5'-CTCGTGCTGATGCTACTGAGGA-3'; p16^INK4A^_Reverse 5'-GGTCGGCGCAGTTGGGCTCC-3', p21_Forward 5'-AGGTGGACCTGGAGACTCTCAG-3'; p21_Reverse 5'-TCCTCTTGGAGAAGATCAGCCG-3'; For analysis of expression of antioxidant and DNA repair associated genes, TaqMan™ Array Human DNA Repair Mechanisms (Cat# 4418773, Thermo Fisher Scientific) and TaqMan™ Array, Human Antioxidant Mechanisms (Cat# 4418764, Thermo Fisher Scientific) were used.

## Trolox equivalent antioxidant capacity (TEAC) assays

TEAC assays were conducted as directed by the manufacturer’s manual.

## Senescence associated β-galactosidase assay

Senescence-associated β-galactosidase activity was assessed according to the manufacturer’s manual (Cat# 9860 and 23,833, Cell Signaling Technology).

## Antioxidant response element (ARE) luciferase assay

To evaluate the NRF2 activation, a stable cell line was generated as previously described (Yang et al., 2019). To evaluate the botanical samples, the cells were seeded (1 × 10^4^ cells/well) in 96-well plates. The cells were treated with the ingredients at indicated concentrations in Fig. 3 and then incubated for 48 h. A luciferase assay kit (Biotium, Inc.) was used to analyze luciferase activity as directed by the manufacturer's manual. Light emission was measured using a microplate reader (Spectramax M5, Molecular Devices, CA, USA).

## In-Cell Western (ICW)

Cells (4 × 10^3^ cells/well) were seeded in a 96-well plate and were treated with carotenoids (lycopene, Lu/Zea, or β-carotene). After 48 h incubation, cells were washed in PBS and fixed in the plate with 4% PFA in PBS for 10 min at RT. After washing with PBS, the cells were permeabilized with 0.2% Triton X-100 in PBS for 15 min and washed three times with PBS. Cells were blocked with blocking buffer (Li-COR) for 90 min at RT, followed by overnight incubation with rabbit anti-OGG1 (Fisher, Cat# PA131402, dilution ratio is 1:200) in blocking buffer including 0.2% tween-20. After three washes with PBST, the cells were incubated with a secondary donkey anti-rabbit antibody (IRDye 800CW) in a blocking buffer including 0.2% tween-20. For normalization, 1/500 dilution of CellTag™ 700 iodide (Li-COR) in blocking buffer including 0.2% tween-20 was used. After 1 h of incubation and three washes PBST, OGG1 was detected using an infrared fluorescent scanner (Odyssey DLx, Li-Cor, Les Ulis, France). Relative fluorescent intensity for OGG1 per cell was divided by vehicle to analyze the percent change in OGG1 intensity relative to the vehicle group. All experiments were repeated in triplicate.

## Isolation of nucleus and cytoplasmic fractions and immunoblotting

The cells were treated with indicated conditions and washed with PBS. Isolation of nucleus and cytoplasmic fractions was conducted as directed by the manufacturer’s manual (ThermoFisher Scientific, Cat.#: 78833). The concentrations of isolated protein were analyzed using a BCA Protein Assay kit (ThermoFisher Scientific, Cat.# 23227) as directed by the manufacturer’s manual. For immunoblotting, the isolated proteins were prepared in β-mercaptoethanol-containing 4 × Laemmli sample buffer (Bio-RAD, Cat.#: 1610747) and were run following immunoblotting protocol described previously (Won et al., 2022). Briefly, the prepared samples were resolved on SDS-PAGE and were transferred to low fluorescence PVDF membrane (Bio-RAD, #1620260). Indicated antibody applied to the membrane and the antibodies are listed in the materials section. The images of immunoblotting were taken using the Odyssey® CLx Imaging System with Image Studio™ Software V. 5.0 (LI-COR). Uncropped images are attached in supplementary information.

## Standard stock solutions

Commercial standards were prepared in Ethyl lactate (lycopene), a 50/50 (v/v) cocktail of MeOH/ MeMT (lutein and zeaxanthin), and in a 50/50 (v/v) cocktail of tetrahydrofuran/ dimethyl sulfoxide (β-carotene) at concentration 1 mg/ml. Solutions were kept at −70 °C.

## Carotenoid quantitation

The extraction and chromatography methods (Cortes-Herrera et al., 2019) were adapted with slight modifications. Quantification of carotenoids by UV (lutein, zeaxanthin, and β-carotene) and mass spectrometry (lycopene) were used in the study. Due to the low UV absorption of lycopene, mass spectrometry was used for the measurement of this carotenoid. The ionization of carotenoids in positive ESI did not lead to the formation of protonated ions (M + H)^1+^, but a molecular cation (M^+.^). This phenomenon was reported previously for lycopene with a poor concomitant fragmentation of such ions (Cortes–Herrera et al., 2019). Moreover, due to the complicated commercial availability of an isotopically labeled standard for lycopene at the time of analysis, the latter was quantified via a standard addition method that enables matrix effect correction (Hasegawa et al., 2021).

## Cell extraction and quantitation

Three ceramic balls and 250 µl of ice-cold ethyl lactate were added into each tube with a cell pellet followed by incubation at – 70 °C for 30 min. Cells were disrupted in TissueLyzer II (Qiagen, Milden, Germany) at the frequency of 15 Hz in two cycles of 7 min each at ambient temperature. Disrupted cells were vortexed for 30 min (Fisher Genie 2) at frequency 7 and centrifuged at 20,000 × g at ambient temperature for 30 min. Supernatants were transferred into clean tubes and stored on ice until further manipulation.

The calibration curve for UV quantification was prepared in supernatants of cells not exposed to carotenoids by twofold serial dilutions on the day of analysis. For the quantification of lycopene, the serial dilutions of the lycopene stock in ethyl lactate were prepared at concentrations tenfold higher than the desired concentrations in samples destined for quantitation. Triplicates of cell extracts exposed to lycopene were combined and then distributed in 90 µl aliquots. The aliquot representing “0” addition received 10 µl of ethyl lactate. Other aliquots received 10 µl of a correspondent standard addition sample to yield the desired concentration.

All test samples and aliquots of the calibration curve were analyzed using an Acquity UPLC H-class chromatograph equipped with a UV–Vis detector (Waters) and connected to Orbitrap Tribrid Fusion (ThermoFisher) mass spectrometer. Samples were loaded (12 µl) onto HALO C-30, 2.1 × 150 mm column packed with 2.7 um particles (Allentown, PA). Separation was executed at 0.25 ml/min and 10 °C using water (A), MeOH (B), and MTBE (C) supplemented with 0.05% (v/v) of FA. The initial, 1st minute gradient of 10% A and 90% B was followed by 0 to 20% gradient of C (90 to 80% B), and A (10 to 0%)) for 27 min. Then, the separation was continued using binary (B and C) solvent combinations: isocratic 20% C between 28 and 33 min, followed by an increase of C from 20 to 27% between 33 and 35 min and a further increase to 37.5% between 35 and 43 min. Then, the solvent C was held constant (37.5%) for two minutes. The gradient was switched to 100% B at the 45th minute and held isocratic for 2 min. Finally, the system was equilibrated in 10% A and 90% B for 18 min.

Data for UV analysis was acquired at 450 nm. The mass spectrometry data was acquired using the HESI 2.0 source in a positive mode (3750 V, sheath gas—10, Aux gas – 6, Ion transfer tube − 250 °C, and Vaporizer − 175 °C). Ions were scanned in the range of 500–600 m/z at the resolution 120,000 (FWHM). Mass spectrometry and chromatography data was used for the confirmation of carotenoids in UV peaks and quantification (peak area) of lycopene at 568.4276 (lutein, zeaxanthin) and 536.4375 (β-carotene and lycopene) with 5 ppm accuracy.

## Statistical analyses

GraphPad Prism 8 (GraphPad Software, San Diego, CA, USA) was used to conduct one-sided, two-sample with equal variance t-test analyses. Data are presented as the mean ± standard deviation. A value of *p* < 0.05 was considered statistically significant. For ICW, immunofluorescence, and senescence-associated β-galactosidase assay, representative images are presented. Each of these experiments was independently repeated at least three times.

## Results and discussion

### Carotenoids reduced BL-induced ROS generation

With the emergence and widespread use of electronic devices in our modern lifestyle, we are constantly exposed to BL. It is known that retinal exposure to excessive levels of BL induces photochemical damage to human RPE (Nunez–Alvarez et al., 2019). Therefore, the BL-induced degeneration of the human RPE presents a more relevant in vitro model for our study than the H_2_O_2_ exposure models.

A compelling body of research showes that dietary antioxidants may play a prophylactic and therapeutic role in a range of ophthalmic disorders associated with oxidative stress. Clinical investigations demonstrate that supplementation with antioxidant nutrients, such as β-carotene, and vitamins C and E, is linked to a reduced risk of AMD (Lin et al., 2009). Furthermore, lutein and zeaxanthin exhibit preferential accumulation in the macula of the retina, where they comprise the macular pigment and offer defense mechanisms to execute a protective function against oxidative damage (Mozaffarieh et al., 2003).

We quantified the carotenoids absorbed in the cells using UV and mass spectrometry (Cortes-Herrera et al., 2019). ARPE-19 cells were incubated for 24 h in the media including vehicle (DMSO: THF) or indicated carotenoids. A 5:1 ratio blend of lutein and zeaxanthin (Lu/Zea) was employed as a singular ingredient. The cells were treated with 10 µg/ml of Lu/Zea, β-carotene (18.6 µM), or lycopene (18.6 µM). After incubation, cells were rinsed 5 times with PBS and analyzed as described in the methods section. The amounts of absorbed carotenoids were 0.73 µg**/**1 × 10^6^ cells for lycopene, 0.37 µg**/**1 × 10^6^ cells for lutein, 0.07 µg**/**1 × 10^6^ cells for zeaxanthin and 0.06 µg**/**1 × 10^6^ cells for β-carotene (Table 1).

**Table 1:** Quantification of absorbed carotenoid in ARPE-19

	Lycopene	Lutein	Zeaxanthin	β-carotene
Amount in cell pellet (µg/1 × 10^6^ cells)	0.73	0.37	0.07	0.06
Relative standard deviation (%)	5.4	8.3	4.7	6.8

ARPE-19 cells were incubated for 24 h in the media containing either vehicle or carotenoids. The cells were treated with 10 µg/ml of lycopene, Lu/Zea, or β-carotene. After incubation, carotenoid content was measured as described in the methods section

**Fig. 1:**
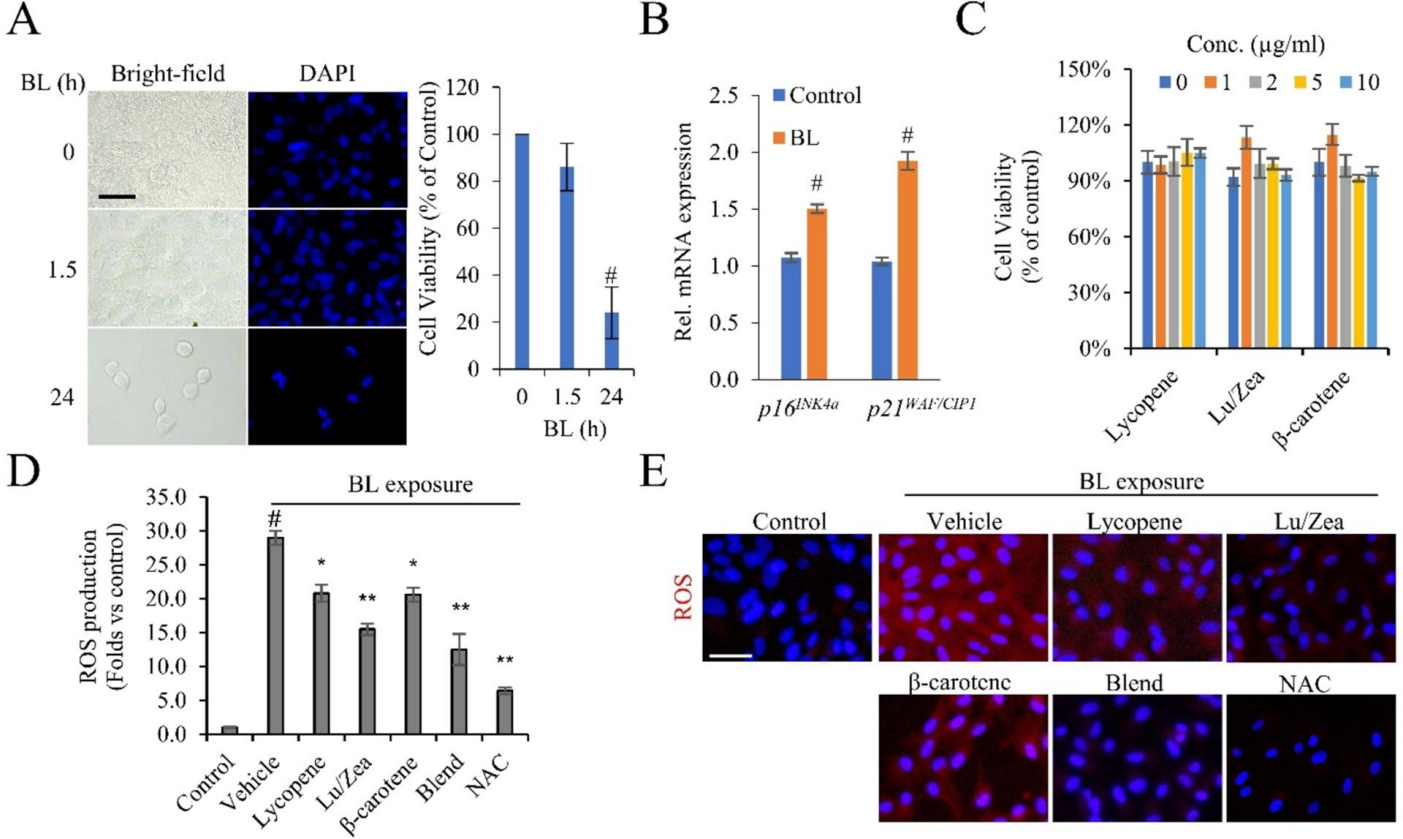
Carotenoids reduced BL-induced ROS formation in ARPE-19 cells The cells were irradiated with BL for 1.5 and 24 h and microscopic pictures were taken to show the cell morphology. **A** After BL exposure, the cells were fixed and stained with DAPI. Scale bar: 50 µm. **B** *p16*^*INK4A*^ and *p21*^*WAF1/CIP1*^ mRNA expression was analyzed by qRT-PCR following exposure to BL using GAPDH as a housekeeping gene. **C** Cells were treated with indicated concentrations of carotenoids (lycopene, Lu/Zea, or β-carotene) for 48 h. Cell viability was analyzed by MTT assay. **D** Cells were pre-treated with the condition described in Methods, and ROS production was analyzed. **E** Blue-light-induced ROS generation was detected by using CellROX™ staining and immunofluorescence microscopy. DAPI was used to counterstain the cell nuclei. Data is presented by mean values ± SD (*n* = 3). Lutein/zeaxanthin (Lu/Zea). #*p* < 0.01 control vs. vehicle; *p < 0.05, **p < 0.01 vehicle vs. treatment

Light induced degeneration of the RPE is a more relevant in vitro model for studies of AMD (Boutzen et al., 2020). We set the conditions of BL exposure to 2.5 mW/cm^2^ for 1.5 h to further examine BL-induced ROS formation in ARPE-19 cells. BL exposure with 2.5 mW/cm^2^ for 1.5 h did not affect cell viability (Fig. 1A) but did induce cellular senescence. After BL irradiation, the mRNA expression of senescence markers, *p16*^*INK4a*^ and *p21*^*WAF1/CIP1*^, were increased (Fig. 1B). In natural dietary sources, lutein and zeaxanthin are found as a 5:1 ratio. (Kotagiri et al., 2022). In this research, to evaluate the optimized antioxidant effect specifically targeted at ocular health, we used the ratio (5:1) of lutein and zeaxanthin (Lu/Zea), along with the carotenoid blend containing lycopene, Lu/Zea, and β-carotene. None of the carotenoids including lycopene, Lu/Zea, and β-carotene showed toxicity in ARPE-19 cells. (Fig. 1C). BL exposure in the untreated ARPE-19 cells increased ROS levels significantly compared to BL irradiated vehicles, but much lower ROS generation was shown in the cells with N-acetylcysteine (NAC) as a positive control. (Fig. 1D, E). Next, we pretreated the individual carotenoids and carotenoid blend in the APRE-19 cells for 48 h and examined ROS generation levels after BL exposure. For the carotenoid blend treatment, we blended the individual carotenoids in a specific ratio. This ratio was determined as follows: Lycopene: Lutein: Zeaxanthin: β-carotene = 5.7:10:2:0.5. Pretreatment with individual carotenoids reduced ROS generation. The carotenoid blend showed the most reduced ROS levels (Fig. 1D, E). These results demonstrated both the antioxidant effect of individual carotenoids as well as the maximizing effect of the carotenoid blend to reduce ROS generation induced by BL exposure in ARPE-19 cells. These findings highlight the potential health benefits of dietary carotenoids in reducing cell damage in human RPE from oxidative stress induced by the exposure to blue light.

## Antioxidant activity of carotenoids

**Fig. 2:**
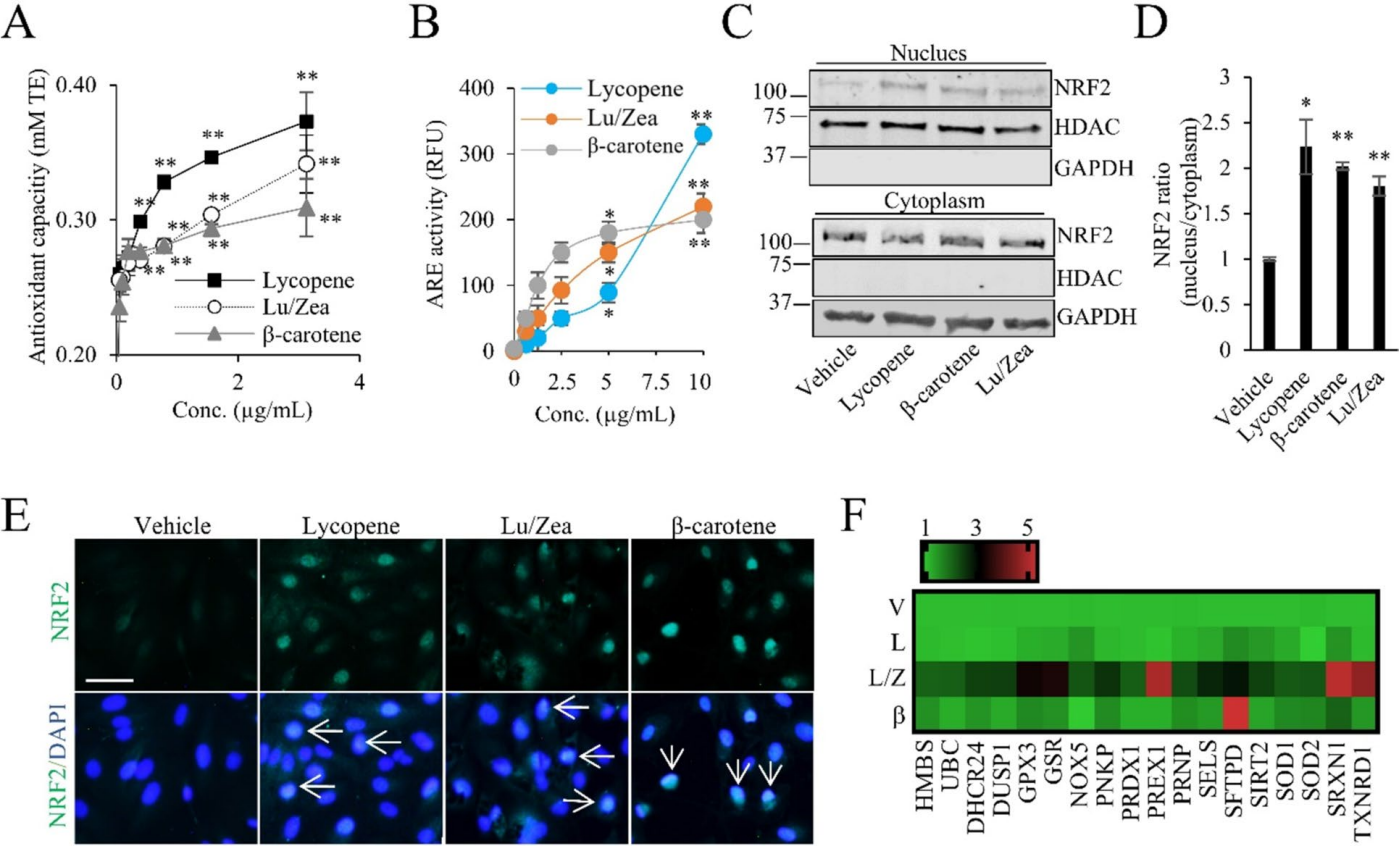
Analysis of antioxidant properties of individual carotenoids. **A** TEAC assay was used to monitor the antioxidant activities of the carotenoids at indicated doses. **B** ARPE-19 cells were incubated with the indicated condition for 48 h and ARE-responsive luciferase reporter activity was analyzed. **C** The cells were exposed to carotenoid solutions (10 µg/ml of lycopene, Lu/Zea, or β-carotene) for 24 h. After the treatment, NRF2 translocation and the mRNA expression of antioxidant mechanism associated genes were analyzed. Nucleus and cytoplasm fractions were isolated and NRF2 levels were proved. **D** NRF2 ratio was analyzed with band density of nucleus and cytoplasmic fractions. **E** Immunofluorescence staining of NRF2 (green) in the cells and nucleus was counterstained with DAPI. Scale bar: 50 µm. **F** Heatmap depicts the relative mRNA expression level of antioxidant mechanism associated genes from indicated groups. All values were normalized to GAPDH expression. Vehicle (V), lycopene (L), lutein/zeaxanthin (L/Z), β-carotene; (β). Images are representative of three independent replicates. Data are shown as mean ± SD (*n* = 3). Significant difference (*p < 0.05, **p < 0.01) was confirmed in comparison to vehicle

We investigated the mechanisms by which carotenoids exert their antioxidant properties. Lycopene, Lu/Zea, and β-carotene showed significant antioxidant activity in a dose-dependent manner (Fig. 2A). These results support carotenoids’ antioxidant potential for scavenging ROS and reducing ROS generation induced by BL irradiation. The antioxidant capacity of the individual carotenoids was investigated using TEAC assay (Bohm et al., 2002). The antioxidant capacity of carotenoids was reflected differently depending on the analysis method and the antioxidant activity of β-carotene is well reflected without degradation to long-chain decomposition products with analysis by TEAC assay (Mueller and Boehm, 2011). In this context, the TEAC assay was adapted to evaluate the antioxidant activity of lycopene and β-carotene as well as Lu/Zea.

## Carotenoids activated the transcription of nuclear erythroid 2-related factor (NRF2)

The transcription factor NRF2 regulates the expression of antioxidative enzymes including glutamate-cysteine ligase catalytic subunit (GCLC), heme oxygenase-1 (HO-1), and NAD(P)H quinone oxidoreductase 1 (NQO1) (Chen and Kunsch, 2004). The expression of the antioxidative enzymes in response to ROS is induced by antioxidant response elements (ARE), which activate the NRF2 (Chen and Kunsch, 2004). ARPE-19 cells were treated with each carotenoid for 48 h and were analyzed for ARE activity. The individual carotenoids each showed a dose-dependent luciferase activity in the ARE reporter assay (Fig. 2B). We evaluated the effect of carotenoids on NRF2 levels within the nucleus using western blotting and immunocytochemistry. It was observed that treatment with individual carotenoids at a concentration of 10 µg/ml of Lu/Zea, β-carotene, or lycopene for 24 h led to increased NRF2 levels in the nucleus (Fig. 2C, D). Analysis of NRF2 expression in the nuclear and cytoplasmic fractions revealed a significant increase (*p* < 0.05) in the NRF2 ratio (nucleus/cytoplasm) by 2.2-fold for lycopene, 2.0-fold for β-carotene, and 1.8-fold for Lu/Zea after treatment (Fig. 2D). The individual carotenoid treated groups showed a notable enhancement in NRF2 levels within the nucleus, as depicted in Fig. 2E. These findings demonstrate that carotenoids enhanced NRF2 levels within nucleus compartments, highlighting their significance in NRF2-ARE signaling pathways. Next, we analyzed gene expressions associated with antioxidant mechanisms. Multiple genes associated with antioxidant mechanisms were significantly increased in Lu/Zea treated cells (Fig. 2F). These results show that carotenoids activated the NRF2 pathway and increased expression of genes associated with antioxidant mechanisms. These results give insight into the carotenoids antioxidant ability to reduce BL-induced oxidative stress.

## Carotenoids reduced DNA damage and cellular senescence triggered by BL exposure

**Fig. 3:**
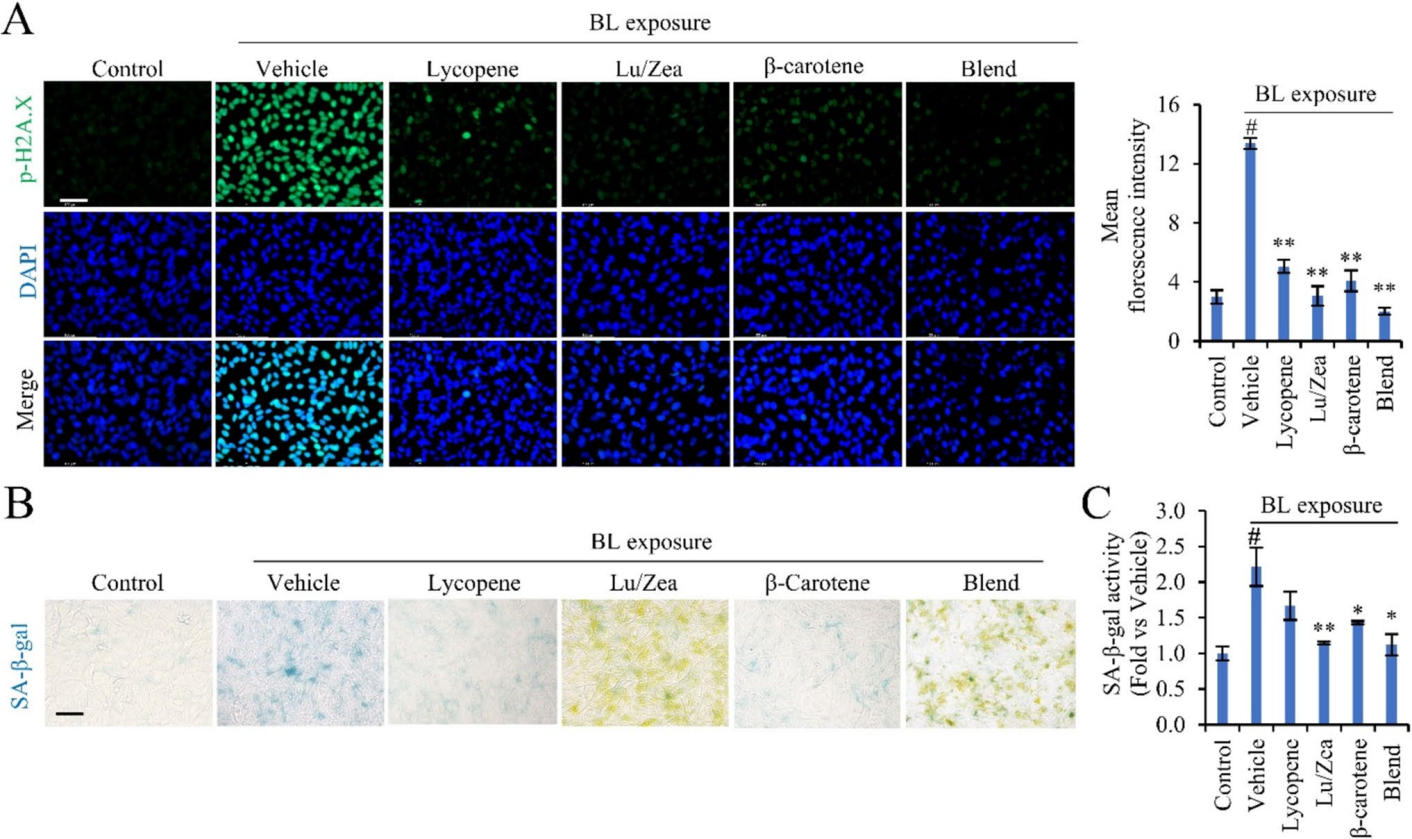
Carotenoids alleviate DNA damage and cellular senescence induced by BL in ARPE-19 cells. Cells were pretreated with indicated carotenoids (10 µg/ml of lycopene, Lu/Zea, or β-carotene) for 48 h. The cells were irradiated with BL. **A** The nucleus was indicated by counterstaining with DAPI (blue) and representative immunofluorescence images were stained with p-H2AX immunofluorescence (green). **B** SA-β-gal stain images and **C** their activity were analyzed in ARPE-19 cells with indicated conditions. After BL irradiation, the cells were maintained in a fresh medium for 24 h. Data are presented by mean values ± SD (*n* = 3). #*p* < 0.01 control vs vehicle; *p < 0.05, **p < 0.01 vehicle vs. treatment. Scale bar: 50 µm, Lutein/zeaxanthin (Lu/Zea)

ROS-mediated oxidative stress leads to DNA damage and serine139-phosphorylated histone H2A.X (p-H2A.X) has been established as evidence of DNA damage (Kurz et al., 2004). We evaluated nuclear p-H2A.X immunofluorescence to assess DNA damage triggered by BL exposure in ARPE-19 cells. BL exposure increased expression of p-H2A.X nuclear foci, which is evident for the activation of the response associated with DNA damage. Pretreatment of cells with individual carotenoids resulted in reduced p-H2A.X expression, while the combination of these carotenoids further decreased p-H2A.X expression (Fig. 3A).

It is known that DNA damage causes cellular senescence (Chen et al., 2007). In instances where DNA damage is excessive or accumulates beyond the capacity of DNA repair mechanisms, cellular senescence will occur (Chen et al., 2007). Next, we analyzed the effects of carotenoids on cellular senescence. BL exposure led to an increase in SA-β-gal expression, indicating higher senescence levels, as well as enhanced SA-β-gal activity but pre-treatment with individual carotenoids showed fewer SA-β-gal positive cells (Fig. 3B) and lower SA-β-gal activity (Fig. 3C). Lu/Zea and the carotenoid blend showed significantly lower SA-β-gal activity, 1.2 and 1.1-fold respectively compared to 2.2-fold of vehicle (Fig. 3C). These results showed carotenoids’ preventive effect against BL-induced DNA damage and cellular senescence in ARPE-19 cells.

Repetitive and chronic non-lethal RPE damage with oxidative stress are regarded as significant factors for the pathogenesis of AMD disease (Cano et al., 2010). ROS are harmful to DNA and a variety of cellular organelles (Algvere et al., 2006). It is known that ROS causes DNA damage and triggers cellular senescence, which might be involved in AMD (Kozlowski, 2012). Here pre-treatment with individual carotenoids reduced DNA damage and cellular senescence triggered by BL exposure in ARPE-19 cells (Fig. 3). Moreover, the carotenoid blend showed a higher effect on lowering DNA damage and cellular senescence as compared to the effect of individual carotenoids. These results demonstrate the protective effect of carotenoids in reduction of DNA damage and cellular senescence triggered by BL exposure.

## Carotenoids increased the expression of DNA repair associated genes

**Fig. 4:**
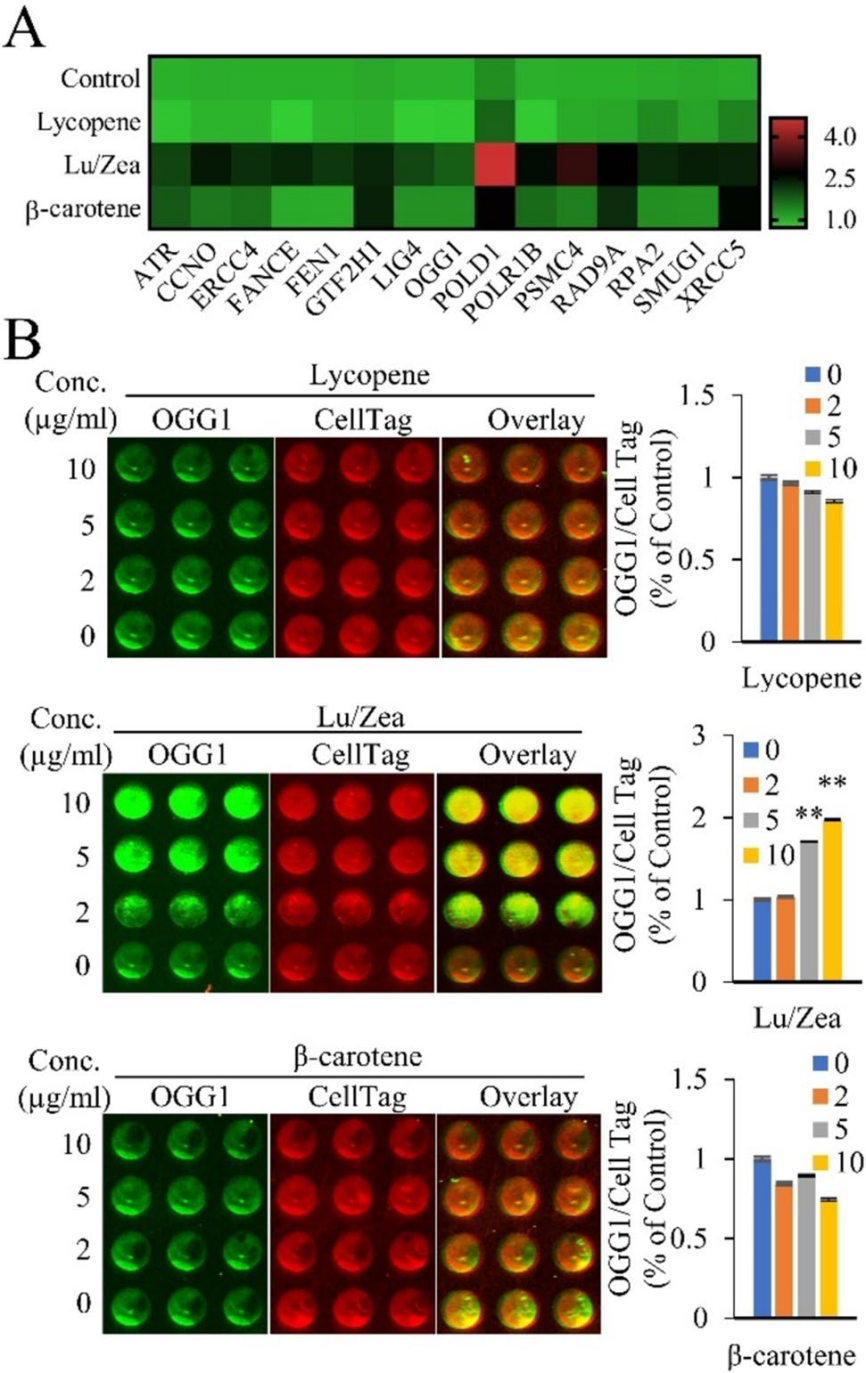
Effects of carotenoids on the expression of genes associated with DNA repair mechanism. The cells were exposed to carotenoid solutions (10 µg/ml of lycopene, Lu/Zea, or β-carotene) for 48 h. **A** Heatmap displays the relative mRNA expression level of DNA repair mechanism associated genes from indicated group. GAPDH was used as a reference gene. **B** Representative images of ICW plates. The cells were exposed to the indicated concentrations of carotenoids for 48 h. Expression of OGG1 was detected as green fluorophores. CellTag was used for cell number normalization and detected as red fluorophores. Quantification of fluorescence signal intensity compared with untreated control and intensity of CellTag was used for normalization. Data is presented as mean ± SD (*n* = 3). A significant difference (**p < 0.01) was observed in treated cells compared to control. Lutein/zeaxanthin (Lu/Zea)

To further explore the underlying mechanisms involved in carotenoids reduction of BL-induced DNA damage, we investigated expression levels of DNA repair associated genes in ARPE-19 cells. Treatment with Lu/Zea or β-carotene significantly increased the expression levels of genes associated with DNA repair mechanisms when compared to the control group (Fig. 4A). Next, we confirmed a significant dose dependent increase in expression of OGG1 protein in Lu/Zea treated cells. (Fig. 4B). The base excision repair (BER) pathway appears as a primary DNA repair mechanism, and the BER pathway is initiated by expression of DNA repair associated genes such as 8-oxo guanine DNA glycosylase-1 (*Ogg1*) in mammals (Wang et al., 2018). It is known that the expression of OGG1 is regulated by NRF2 pathway (Singh et al., 2013). Induction of NRF2 is associated with the inhibition of DNA damage via up-regulation of OGG1 (Singh et al., 2013). OGG1 is the major DNA repair glycosylase and deficiency of OGG1 has been implicated in aging and AMD (Jarrett and Boulton, 2012). Taken together, these results showed that Lu/Zea and β-carotene can influence DNA repair by modulation of expression of DNA repair associated genes, that provide a potential protective mechanism against a BL-induced cellular damage.

## Carotenoids reduced secretion of IL-6 and VEGF after BL irradiation

**Fig. 5:**
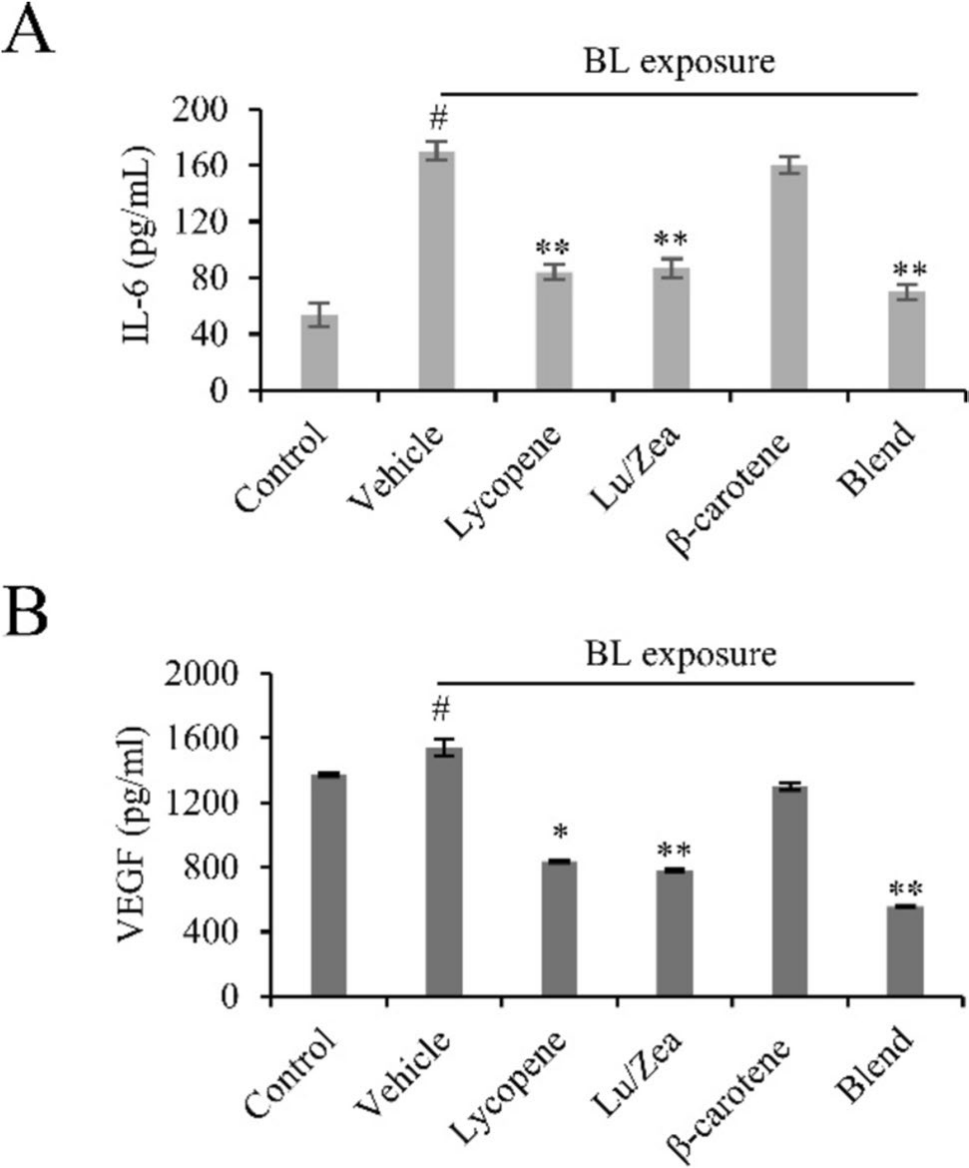
Effect of carotenoids on the production of IL-6 and VEGF. Cells were pretreated with indicated carotenoids (10 µg/ml of lycopene, Lu/Zea, or β-carotene) for 48 h. After BL irradiation the cells were incubated in a fresh medium for 24 h. IL-6 (**A**) and VEGF (**B**) secretion levels were measured in the supernatants. Data are shown as mean ± SD (*n* = 3). #*p* < 0.01 control vs. vehicle; *p < 0.05, **p < 0.01 vehicle vs. treatment. Lutein/zeaxanthin (Lu/Zea), Blend (combination of Lycopene, Lu/Zea, and β-carotene)

One of the major cytokines (IL-6) in the senescence-associated secretory phenotype (SASP), is known to be involved in the development of AMD (Seddon et al., 2005). In our research, the release of IL-6 was analyzed after BL irradiation in the control ARPE-19 cells. Secretion of IL-6 was increased significantly after BL irradiation (Fig. 5A). After pre-treatment with lycopene or Lu/Zea there was a significant decrease in IL-6 secretion as compared to the vehicle. The carotenoid blend showed the highest reduction of IL-6 secretion compared to individual ingredients (Fig. 5A).

RPE naturally secret vascular endothelial growth factor (VEGF) (Miller et al., 2013). VEGF signaling regulates normal vascular development, but it is also known that overexpression of VEGF is involved in pathological angiogenesis in neovascular AMD (Miller et al., 2013). We examined whether the carotenoids regulated the synthesis of VEGF in ARPE-19 cells. Following BL exposure, the secretion of VEGF protein levels was increased significantly. However, pre-treatment with individual carotenoids maintained significantly lower levels of VEGF (Fig. 5B). The carotenoid blend showed a higher effect on inhibition of VEGF release compared to individual carotenoids (Fig. 5B). Taken together, these findings demonstrate that both individual carotenoids and combination suppressed the secretion of both IL-6 and VEGF, potentially mitigating the pathological features associated with AMD.

Our findings demonstrated that BL irradiation triggered DNA damage and cellular senescence in ARPE-19 cells, which enhanced IL-6 and VEGF secretion. Senescent cells may initiate the signs of AMD pathogenesis: inflammation, and activation of pro-angiogenic cytokines. Ocular anti-VEGF therapy occupies a pivotal position in treating sight-threatening ophthalmic conditions such as diabetic retinopathy and neovascular AMD (Sene et al., 2015). This novel work supports that supplementation with dietary carotenoids can promote multiple mechanisms for protection of human RPE against BL irradiation. Additional comprehensive studies of the senescent response in the RPE are needed to further elucidate the development of AMD and to comprehend preventive and therapeutic modalities. In summary, carotenoids, as potent antioxidants, have the potential to enhance protective mechanisms against oxidative stress damage induced by BL exposure. The carotenoids promote the NRF2 translocation levels to the nucleus and activate the ARE pathway. Furthermore, carotenoids also mitigate DNA damage and cellular senescence, possibly through DNA repair mechanisms. Carotenoids demonstrated a potential to prevent the onset and progression of AMD by decreasing the secretion of IL-6 and VEGF, pathological features associated with AMD. Consequently, carotenoids emerge as crucial and essential components in preserving eye health from the deleterious effect of excessive BL exposure.

## Supplementary Information

Below is the link to the electronic supplementary material.

Supplementary file1 (PDF 74 kb)
