# Supplementary figures and images for: Protective effects of carotenoids against blue light induced-cellular damage in human retinal pigment epithelium
Source: Food Sci Biotechnol. 2025 Jan 27;34(8):1713–23. doi: 10.1007/s10068-024-01757-z (PMC11936865; doi:10.1007/s10068-024-01757-z)

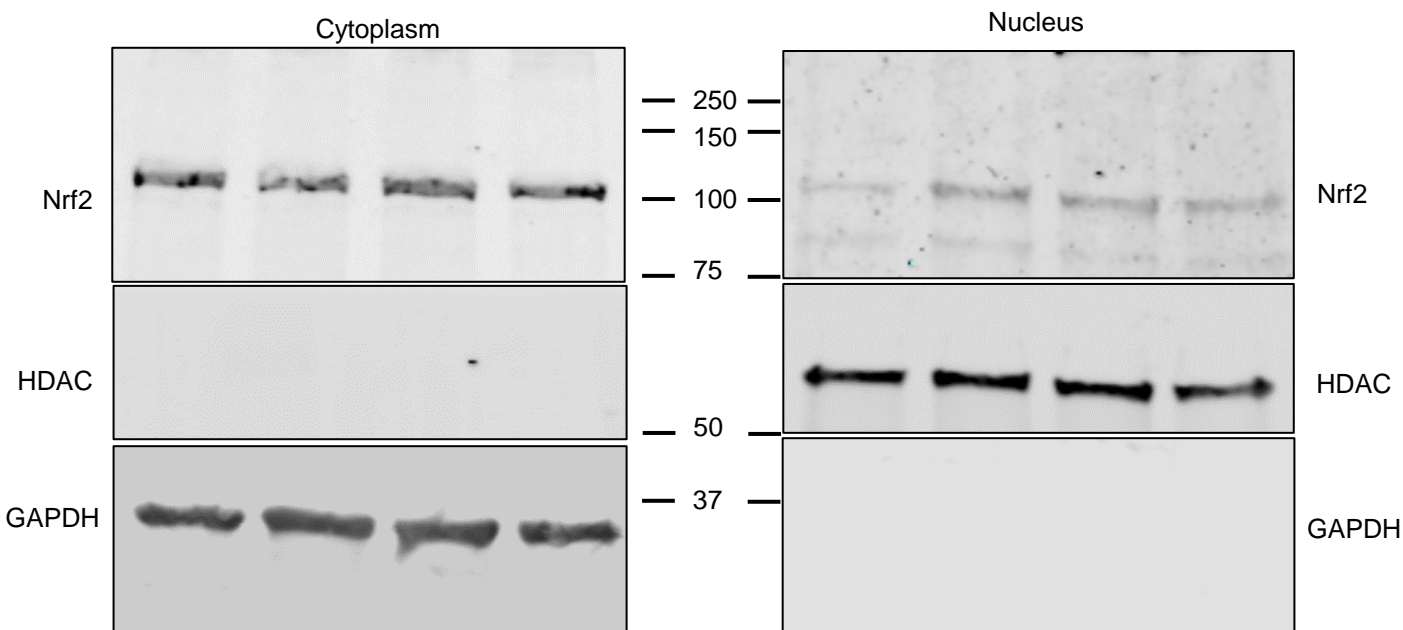

Supplement: Supplementary file 1 — Supplementary file1 (PDF 74 kb) [file 10068_2024_1757_MOESM1_ESM.pdf]
